# Optimization model for production scheduling taking into account preventive maintenance in an uncertainty-based production system

**DOI:** 10.1016/j.heliyon.2023.e17485

**Published:** 2023-06-21

**Authors:** Plamen Penchev, Pavel Vitliemov, Ivan Georgiev

**Affiliations:** a Department of Business and Management, University of Ruse, Studentska 8, 7017, Ruse, Bulgaria; b Department of Applied Mathematics and Statistics, University of Ruse, Studentska 8, 7017, Ruse, Bulgaria; c Institute of Mathematics and Informatics, Bulgarian Academy of Sciences, Department of Information Modeling, Acad. Georgi Bonchev Str., Block 8, 1113, Sofia, Bulgaria

**Keywords:** Scheduling, Production planning, MILP, Optimization model, Uncertainty, Maintenance

## Abstract

In the dynamic yet uncertain environment of Industry 4.0, industrial companies are utilizing the benefits of contemporary technologies in manufacturing by striving to implement optimization models in each stage of the decision-making process. Many organizations are focusing particularly on the optimization of two key aspects of the manufacturing process - production schedules and maintenance plans. This article presents a mathematical model with the main advantage of finding a valid production schedule (if such exists) for the distribution of individual production orders on the available production lines over a specified period. The model further considers the scheduled preventive maintenance activities on the production lines, as well as the preferences of the production planners regarding the start of the production orders and non-use of certain machines. When necessary, it also offers the possibility to make timely changes in the production schedule, and thus to handle the uncertainty as precisely as possible. For the verification of the model, two experiments were conducted (quasi-real and real-life), with data from a discrete automotive manufacturer of locking systems. The results from the sensitivity analysis demonstrated that the model further optimizes the execution times of all orders, and specifically the production lines usage - their optimal load and non-use of unnecessary machines (valid plan with 4 out of 12 lines not used). This allows for cost savings and raises the overall efficiency of the production process. Thus, the model adds value for the organization by presenting a production plan with optimal machine usage and product allocation. If incorporated into an ERP system, it could distinctly save time and streamline the production scheduling process.

## 1 Introduction

In today's dynamic market environment, industrial companies have to keep themselves agile all the time. Often they are using advanced production plans and schedules, which consider the status of operations and machines on the manufacturing shop floors. Using real-time data allows production managers and operation planners to adapt the manufacturing systems to highly dynamic and unexpected disruptive events, to prevent interruption of the production process. Such reactions are required due to unforeseen events and may include accelerating or delaying an operation, changing the starting time of maintenance actions, delaying, speeding up or rerouting jobs and operations. Concepts of Industry 4.0 such as cyber-physical systems, the Internet of things (IoT), operations, equipment, detailed machine information, orders and work-in-progress provide the necessary real-time data that are integrated into a company's business software for resource planning, maintenance management and material requirements. This makes the information constantly available for necessary decision-making, including maintenance planning and production scheduling, all of which are crucial elements of any manufacturing process [1,2].

There are studies focused on flexible job shop scheduling. The majority of them considered the production environment to be static (i.e. all required data are available at time zero and are not subject to change with time) and the machines to be always available for production (i.e. never break or undergo planned maintenance). In practice, however, manufacturing systems go through interruptions and unexpected events that are stochastic. They also cope with the uncertainty of production environments such as due date fluctuations, order cancellations, new orders and changes that may result from the customers’ preferences, material shortages, unexpected machine breakdowns and (un)scheduled maintenance activities. All of these factors force the production planners to make frequent updates to the production plans to react adequately to these changes [3].

The preventive maintenance in the area of Industry 4.0 is part of the performance enhancement and cost reduction processes of the production systems, as it detects early problems in machinery operations by analyzing large volumes of historical and real-time data. It may also predict future failure modes, improve machine life expectancy and create maintenance plans for lessening or preventing the influence of anticipated breakdowns [4,5].

In the operation management strategy, several studies are focused on the importance of the joint optimization of maintenance planning and production scheduling. Undoubtedly, production planning models which jointly optimize maintenance plans and production schedules are crucial in practice. They elevate the importance of research investigating the joint optimization of maintenance, production planning and scheduling in flexible flow shops with real-time dynamics, including due date changes, machine degradation, new job arrivals, minimal repairs and machine breakdowns [6].

Models for optimal scheduling in manufacturing systems were presented by different authors [7,8]. However, the technical literature lacks suitable models for the production scheduling optimization of manufacturers with flexibility in production lines. Such models would enable the producers to modify their operation schedules and optimally allocate the available resources to fulfill the time-varying demand for different products. Furthermore, taking into account the preventive maintenance and repair policy at all times, the models would help to find an optimal sequence for the working process.

This paper introduces an optimization model for production scheduling taking into account preventive maintenance in an uncertainty-based production system that operates under changing and unexpected events. The uncertainty of the production environment has numerous expressions. Among them are the frequent new job arrivals with fluctuating parameters (e.g. changing quantities); due date modifications (e.g. earlier demands); machines wear, unforeseen breakdowns and downtimes; delays in single components and raw materials supply, as well as quality issues with them (affecting the scheduled production start); unpredicted drops in the availability of required direct workers and indirect employees.

The proposed optimization model can serve as a tool for production planners and manufacturing managers in devising a more efficient production schedule. Tested with an example from real-world manufacturing, the model can add value by presenting a solution for the preparation of the production plan with optimal machine usage and product allocation. If required, the model is also capable of processing immediate changes in the production schedule, reflecting the potential challenges from the uncertain manufacturing environment, and thus handling the uncertainty as precisely as possible.

The article is organized as follows: Section 2 describes the problem. Section 3 formulates the mathematical model and develops a solution method for the proposed model. The numerical experiments and results are presented in Section 4. Section 5 discusses the results and the benefits of the model. Section 6 concludes the paper with a summary of our contributions, specifying the limitations of the study and research propositions.

## 2 Problem description

### 2.1 Production planning

The overall performance of the production system depends to a large extent on the effective production planning and scheduling. Among the main influencing factors are the planning of the production and maintenance and quality management [9]. At the same time, growing customer expectations and technological developments are increasing the complexity of production systems. In addition, manufacturing companies face various disruptions and cyclical requirements in an uncertain environment, which could lead to unbalanced use of their production capacity. Thus, efficient and effective production planning becomes a key competitive advantage [10].

Production planning is one of the main elements of a company's production program and is responsible for ensuring the availability of all production materials and components. It defines the necessary production technоlogies and strategies, as well as the sequence of production steps, in order to enable the operations to be carried out according to pre-defined schedules at the lowest possible cost [10]. Production scheduling is dynamic and always remains “flexible”, as plans may need to be modified depending on changes in the uncertain environment. Among the important issues are.

1. What production capacities are needed (which machines) for the given period?
2. How should they be used to produce the desired products at the desired production rates (which products, when, on which machines)? [11].

The existing main types - production planning (medium/long-term tactical planning) and operational planning (production scheduling) - are two closely related activities that belong to different levels of decision-making in the production system. Higher level production planning determines product stocks in accordance with customer requirements for a long period of time – usually from several months to a year. It is performed, for example, on a monthly basis. Lower-level operational planning, expressed through the production schedule, covers a short horizon of a few days to weeks. It focuses on how to process different products, e.g. the assignment of production tasks to the proper machines and the sequence and time for the execution of the tasks on each machine. In general, the goal of production planning is to fulfill customer orders with minimal production and warehousing costs, while operational planning (scheduling) aims to refine the production tasks coming from the production planning and to minimize their execution time [12,13].

Production plans are usually guided by production objectives and rules in order to improve the company's performance indicators. However, due to the dynamic nature of the production environment, an effective rule in one situation may be ineffective in anоther and may nоt be optimal for an extended period of time. At the same time, production planning systems mainly deal with production capacity and priorities. Problems and situations that are more urgent to resolve than others at the moment arise with varying frequency. This requires companies to periodically adjust their production rules, priorities and planning in order to cope with such sudden changes in the production environment [14].

In general, in the context of increasing global competition, short deadlines and high reliability in meeting the deadlines are becoming central factors for differentiation. In particular, in customer-oriented production, the production planners need to make sure that all orders, essential parts and materials for the production of the company's products are planned for the machines in such a way as to meet the customers' deadlines. A number of other factors must be taken into account, such as the times for setting up and preventive maintenance of the machines, transportation, waiting and accumulated orders, current capacity, etc. In particular, reducing lead times results in increased capacity and flexibility requirements in production [9,12].

Joppen et al. [15] divided the areas of action in terms of production planning into: products, processes and resources. The starting point was usually the products in terms of customer requirements. Numerous variations of the manufactured products and short deadlines for their implementation and delivery were often needed. These varieties complicated the production planning, which sometimes led to a lack of medium and long-term schedules and capacity planning. This in turn resulted in frequent short-term changes in the production program and thus to inefficient processes. Such changes in the production plan did nоt provide, for example, the time needed to set up and maintain the machines, which would lead to inefficiencies in the use of resources [15].

However, most production planning systems in manufacturing companies do nоt include preventive maintenance activities. This could lead to inefficient production processes due to unpredictable machine downtime, execution time fluctuations and numerous urgent orders. Although the strong interaction between production planning, maintenance and quality management has been proven, many planning processes are implemented without a holistic approach that takes these three areas into account. The result is unrelated maintenance and production plans. Unscheduled times of preventive maintenance significantly affect the productivity of the production system. At the same time, short-term changes during production processes are among the main causes of shocks in production. Many of these changes are the result of unplanned machine downtimes, which significantly limits the required flexibility of the production system. As a result, delays that are difficult to compensate for occur, and incur additional costs (for example, due to poor product quality or overtime) [9].

### 2.2 Existing methods for production planning optimization, accounting for maintenance

There are various methods for optimizing the production scheduling. Some of them include algorithms for optimizing the sequence of execution of orders such as the shortest working time, the shortest total processing time or the earliest completion date [15]. However, in order to increase the flexibility and quality of production planning, operational planning optimization models should include an approach to integrate preventive maintenance strategies.

The performance of the manufacturing system depends to a large extent on the successful process planning and design of the shop floor. Among the main influencing factors are 1) Production planning, 2) Maintenance planning and 3) Quality management [9]. Several literature sources proved the strong interaction of these disciplines in terms of productivity, product quality and total costs of the production system. In recent years, various problem-solving approaches were developed to assist the production planning in relation to different targets. Based on the above mentioned three main influencing factors, the existing models can be distinguished into two main groups.

1) Integrated models (in maintenance planning and quality management; and in production planning, quality management and maintenance): They focus at optimizing two or more elements simultaneously.
2) Interactive models (in production planning and maintenance): Their purpose is to optimize a particular function when considering others [9].

#### 2.2.1 Integrated models in maintenance planning and quality management

An optimization model was developed that considered product quality [16]. A monitored system consisted of a fault-prone facility whose state could be described as either “in control” or “out of control”. As soon as the condition of the machine was “out of control”, the requirements regarding product quality could not be met. Deterioration of the facility was described by the Weibull function - maintenance measures were assumed to be imperfect and therefore improved the facility by a certain percentage. The model attempted to find the optimal trade-off between (i) frequently performed maintenance measures that led to a reduction in quality-related costs but increased maintenance costs, (ii) infrequently performed maintenance measures that caused an increase in quality-related costs and (iii) manufacturing defective parts that increased production costs.

#### 2.2.2 Integrated models in production planning, quality management and maintenance

Matyas et al. [17] developed a procedural approach to preventive maintenance planning by making conclusions based on the interaction between condition- and load data, historical quality- and machine data, and medium-term production planning. By interconnecting data from production planning, condition monitoring and process data, as well as historical machine quality- and failure data, it was possible to achieve the best possible product quality, optimized plant availability and reduced maintenance costs. This approach was applied in a production line maintenance control center to predict failures and reveal quality deviations in real time. The methodology was supported by dynamic calculation of the remaining useful life of the machine components, and by data analysis and simulation of causes and effects.

#### 2.2.3 Interacting models in production planning and maintenance

The problem of incomplete maintenance operations can be solved by the non-linear mixed-integral optimization model of Aghezzaf et al. [18]. A specific machine, on which corrective and preventive maintenance operations are performed, was monitored. It was assumed that the state of the machine could be determined stochastically and therefore the number of required maintenance operations within a given planning period could be calculated based on the age of the system. As additional constraints in production planning, the model took into account the number of required maintenance operations and the condition of the machine, regarding the reliability of system. A tool was also available to measure the effectiveness of this heuristic approach, which could be integrated into the ERP systems.

Zhou et al. [19] presented a dynamic-opportunistic maintenance model for a multi-component system in which maintenance planning was able to respond to short-term changes in production order sequences. Short-term changes in the production schedule could not be avoided because of market fluctuations and thus may lead to prioritization or postponement of preventive maintenance operations. System components interacted and supported with each other, resulting in a preventive maintenance operation causing the entire system to shut down. When this condition occurred, the model proposed extra required system maintenance operations to ensure that the opportunity costs from the maintenance operations are as low as possible.

Wong et al. [20] presented a manufacturing process with heterogeneous machines which had distinct demands on maintenance operations — machines required different maintenance measures that led to different downtimes. Also, the history between measures varied among the machines. Using a genetic algorithm, this difficulty was mapped and the cycle time was minimized. The model assumed that maintenance actions were “flawless”, meaning that the machine state was set to “new” after a maintenance measure was carried out.

Another optimization model specifically aimed at the difficulties of interconnected facilities - if a facility is damaged due to a preventive maintenance operation, the entire production line is at a standstill [21]. The model aggregated the necessary maintenance measures of different machines, as a result of which to a certain degree they could be carried out simultaneously. The effect of the cumulative measures was subsequently compared with the effect of the measures taken sequentially. The goal was overall reduction in production costs, preventive maintenance costs, repair costs and opportunity costs, caused by process operations delays.

### 2.3 Areas for improvement of the existing methods

Nevertheless, the critical analysis shows that the existing models can be further improved in certain areas so that they achieve even better applicability and the desired optimization for operational planning in real production environment, as well as to obtain more realistic results. Many optimization models normally use a deterministic and a simplifying approach, which may lead to a discrepancy between the stochastic problem and the deterministic solution. And namely the lack of adequate knowledge about the stochastic problem is often the root cause for various problems which the production planners encounter when the production schedule becomes unfeasible under the occurrence of unpredicted events [22].

Some of these potential areas for improvement can be summarized as follows.

1) The holistic optimization and interaction of production scheduling and preventive maintenance is not always fully considered in the existing models.
2) Often the scheduling algorithms compute the maintenance dates based on stochastic, theoretical methods (e.g. Weibull distribution), which rarely take into account the contemporary knowledge about preventive maintenance. Therefore, the actual condition of the machines is not monitored.
3) The substantial computational power of the optimization models often presents a challenge for their integration into the ERP systems. Among the reasons is the large number of planning parameters covered by the approaches. Even though the optimal setting of planning parameters has a significant impact on the operation of the production system, planning systems with a smaller number of parameters are generally easier to work with in practical implementations [23].
4) A lot of models are based on assumptions that do not actually occur in realistic manufacturing environments. Therefore, the validation of many of these models was performed by numerical examples. Practical examples quantifying the advantages for the organizations were often lacking.
5) There is no general validity of the developed models. In many methodologies certain unique framework conditions were considered. However, it is hardly possible they to be generalized to other environments [9].
6) Often the constraints and dependencies among the different machines are disregarded. Many models considered only single production lines, not taking into account a combination of parallel and serial interconnected machines.

### 2.4 Advancements of the proposed optimization model

Our optimization model demonstrates that it is possible to achieve advancements in most of the above-mentioned areas for improvements, building on and extending the knowledge in the following ones.

1) and 2) Our model considers the preventive maintenance activities on the production lines, which are preliminarily scheduled for a certain point of time and for a specific duration. The real condition of the machines is constantly known - they are fully operational throughout the scheduling period, apart from the times of the planned repairs.
3) The number of covered planning parameters is concise – products to be produced, machines available for their production, time slots for preventive maintenance, products' production times (duration, start and end times), production time windows for the machines, and weights of products. This, together with the programming product Matlab, used in creating the model, allows for potentially easier integration with ERP systems (if the code is re-written for the contemporary programming language Python).
4) The model performed a numerical and yet practical example from a real-life manufacturing environment, which quantifies the benefits for the organization. It constructs a production plan with as little as possible number of machines, specifying the exact ones which could be excluded from the schedule. Thus, allowing for numerous associated cost optimizations.

Overall, our developed mathematical model can be utilized in finding viable solutions in optimizing the production schedules – offering optimal machine usage and product allocation, and allowing the manufacturers to make timely changes in the production schedule in response to unpredicted events.

## 3 The proposed real-time production scheduling model

### 3.1 Purpose and requirements

The following real-life constraints in production and production resources have been taken into account in building our optimization model for production scheduling, including preventive maintenance.

1) Constraints in production - Date of export of the manufactured product; Time required for the production of the final product; Machine downtime; Scrap/rework; Availability of single components/raw materials and lead time of suppliers; Overtime work - legally permissible and economically feasible; Coverage days (stock level); Transit time from the factory to the customer - regular or emergency transports.
2) Production resources/machines - Capacity/machine production norm; Types of finished products produced; Productivity of a given final product on a given machine; Necessary prevention - planned maintenance of machines.

In view of the above features of the existing production planning systems, the purpose of our proposed optimization model is.

1) To find possible implementation of the operational schedule and a valid production plan - taking into account all production constraints - all products planned for production in the designated scheduling period to be effectively distributed among the appropriate available machines.
2) To achieve optimization in the execution times of all orders for the planned period (as early as possible), as well as in the use of machines - their full load and non-use of unnecessary machines.

In our constructed mathematical model, it is assumed that a given production company produces n number of products. For each of them, the earliest time at which the production of the product can begin is known, as well as the final moment at which it must be completed. For the production of these products, m number of machines (production lines) are available. In the general case, due to the inhomogeneity of the products, a specific product can only be produced on some of the machines. It should be borne in mind that even if a specific product can be produced on more than one machine, the production time of these machines for a particular product will be different due to the technical specification of each machine. It is also necessary to take into account the technical time required to reconfigure a production line, when it must start production of a certain product. This time is unique and depends on both the machine itself and on the specific product that will be produced. It may also be preferable for certain products to be produced as early in time as possible, while for others it is preferable produce them later or at the last possible moment. The main goal is to find a valid plan as for which product, on which machine and at what time to start production.

The above listed circumstances can be summarized as requirements.

1) for the execution of orders and material base for their implementation - products which must be produced and machines for their production;
2) for timelines - the production of a product cannot start before a certain moment and must end prior to another moment;
3) for compatibility - a specific product cannot always be produced by each machine, but there must be at least one production line that can produce that product;
4) for production time - if the same product can be produced on two or more production lines, then the production time of this product is generally different, depending on which machine the product is produced at;
5) for time setting - the time for setting up a machine for the production of a given product is different and depends on both the machine itself and the type of specific product. It can be taken into account that if two identical products are to be produced one after another on the same production line, the set up time can be neglected (the machine is not reconfigured).
6) for preferences - certain products can be given priority (weight) when starting their production, i.e. production of some products should start as early as possible, whereas of others as late as possible.

Regarding point 6), from a practical point of view it is appropriate the time of production start of each product to be as close as possible to the beginning of its possible time window and to be within that time window. This generally provides more time in case of unforeseen situations - machine damage, problems with the availability of raw materials and other components for the products production, change in the parameters for the execution of certain orders - the need for earlier production, change in the quantity of the produced product, etc.

### 3.2 Methodology

The following notations are used in the developed mathematical model:

(1) m–numberofmachines(productionlines)

(2) n–numberofproducts

(3) $$t_{i}^{1}\;–\;the\;earliest\;possible\;moment\;when\;the\;production\;of\;i^{th}\;product\;can\;start,i=\bar{1,..n}$$

(4) $$t_{i}^{2}\;–\;the\;latest\;possible\;moment\;when\;the\;production\;of\;i^{th}\;product\;can\;start,i=\bar{1,..n}$$

(5) $$t_{ir}\;–\;duration\;of\;production\;of\;i^{th}\;product\;at\;r^{th}\;production\;line,i=\bar{1,..n};\;r=\bar{1,..m}$$

(6) $$p_{ijr}\;–\;technical\;time\;required\;for\;\text{setting}\;\text{up}the\;production\;of\;j^{th}\;product\;manufactured\;on\;r^{th}\;production\;line,provided\;that\;prior\;to\;it\;on\;the\;same\;r^{th}\;production\;line\;is\;produced\;i^{th}\;product\;i,j=\bar{1,..n};\;r=\bar{1,..m}$$

(7) $$\tau_{r}^{1}-a\;moment\;of\;time\;before\;which\;r^{th}\;production\;line\;cannot\;be\;used$$

(8) $$\tau_{r}^{2}-a\;moment\;of\;time\;after\;which\;r^{th}\;production\;line\;cannot\;be\;used$$

(9) $$w_{i}\;–\;weight,giving\;priority\;for\;the\;production\;of\;i^{th}\;product,i=\bar{1,..n}$$

(10) $$T_{i}\;–\;the\;moment\;when\;the\;production\;of\;i^{th}\;product\;starts,i=\bar{1,..n}$$

(11) $$x_{ir}=\left\{ \begin{array}{c} 1,if\;i^{th}\;product\;is\;produced\;on\;r^{th}\;line\\ 0,if\;i^{th}\;product\;is\;not\;produced\;on\;r^{th}\;line \end{array}\right.\;i=\bar{1,..n},r=\bar{1,..m}$$

Equations (1)–(9) are notations of the known input parameters in the model. (10) and (11) are the unknown parameters that we are looking for, with (10) being the production start time of the $i^{th}$ product. Equation (11) is a binary variable, indicating whether the $i^{th}$ product is produced on the $r^{th}$ production line, i.e. which product is manufactured on which machine.

The following clarification should be made regarding requirement 3): If a product i cannot be produced on a production line r, it is presented in the model as $t_{ir}=\infty$, i.e. an infinitely large production time (or a finite number exceeding many times the parameters in the problem) is given for the specific product on the given production line, which effectively prohibits the production of $i^{th}$ product at $r^{th}$ production line.

If the moment $T_{i}$ for the start of production of a specific product is targeted to be as late as possible, then the weight of priority for it $w_{i}$ will be given as negative value.

The following points are unknown at this stage - $T_{i}$ , the time at which production of the products is to begin, and the production lines on which they are to be produced - $x_{ir}$.

The problem is modeled as a partially integer optimization problem with an objective function that the sum of the weighted product production start times is minimal [24,25]:

(12) $$\min Z=\sum_{i=1}^{n}{w_{i}T}_{i}$$

Equation (12) is the objective function in the model, indicating whether we want the production of a product to start earlier or later.

The constraints are:

(13) $$t_{i}^{1}\leq T_{i}\leq t_{i}^{2},\forall i=\bar{1,..n}$$

(14) $$\sum_{r=1}^{m}x_{ir}=1,\forall i=\bar{1,..n}$$

If two products are produced on one production line, only one of the following two constraints must be satisfied:

(15) $$T_{i}+t_{ir}+p_{ijr}\leq T_{j}+\left(2-x_{ir}-x_{jr}\right)M,\forall i,j=\bar{1,..n},\forall r=\bar{1,..m}$$

(16) $$T_{j}+t_{jr}+p_{jir}\leq T_{i}+\left(2-x_{ir}-x_{jr}\right)M,\forall i,j=\bar{1,..n}\text{,}\forall r=\bar{1,..m}$$

and also the following three constraints related to the production line time window must be satisfied:

(17) $$T_{i}\geq \tau_{r}^{1}+\left(1-x_{ir}\right)M,\forall i=\bar{1,..n},\forall r=\bar{1,..m}$$

(18) $$T_{i}\leq \tau_{r}^{2}+\left(1-x_{ir}\right)M,\forall i=\bar{1,..n},\forall r=\bar{1,..m}$$

(19) $$x_{ir}\in \left\{0,1\right\},T_{i}\in R^{+},M\gg 1$$

Constraint (13) is imposed in order to meet the time window for the production of each product.

Constraint (14) ensures that a product can be produced on exactly one machine, i.e. the product cannot be split into two or more parts.

Constraint (15) specifies that if the start of production of the $i^{th}$ product is before that of the $j^{th}$ product, then the sum of the production start time $T_{i}$, the production duration $t_{ir}$ and the setup time $p_{ijr}$ (for the production preparation of the next $j^{th}$ product on the same $r^{th}\;production\;line$), must be before the production start time $T_{j}$ of the $j^{th}$ product.

Similarly is the interpretation of constraint (16), but vice versa in this case the production of the $j^{th}$ product precedes that of the $i^{th}$ product.

Constraints (17) and (18) are related to the fact that if the $i^{th}$ product is produced on $r^{th}$ line, then the time of starting production must be within the time window of that machine.

In constraints (15)–(19) for the value of M a very large positive number is set: M≫1. If these two activities are performed on the same production line, the binary variables $x_{ir}$ and $x_{jr}$ would simultaneously take the value 1 and the equation $\left(2-x_{ir}-x_{jr}\right)M$ in (15), (16) would cancel each other out, resulting in these constraints becoming active. If these activities are carried out on different machines, then at least one of the variables $x_{ir}$, $x_{jr}$ will cancel out and constraints (15) and (16) will always be satisfied i.e. there is no need for them since the products are produced on different machines. Similarly is the interpretation of M in (17), (18).

The problem that arises in model (12)–(19) is that if two products are produced on the same production line, exactly one of the two constraints (15) or (16) must be satisfied for the corresponding indicators. This leads to nоn-convexity of the admissible region and so the problem posed is not linear. This drawback can be fixed by introducing additional unknown binary variables [26,27]:

(20) $$y_{ij}=\left\{ \begin{array}{c} 1,if\;the\;production\;start\;of\;i^{th}\;product\;preceeds\;that\;of\;j^{th}\;product\\ 0,if\;production\;start\;of\;i^{th}\;product\;{doesn}^{\prime }t\;preceed\;that\;of\;j^{th}\;product \end{array}\right.\;i,j=\bar{1,..n}\text{.}$$

Constraint (20) is an additional (auxiliary) binary variable, indicating whether the production start of a given product precedes in time the start of another product.

Then constraints (15) and (16) will be modified as follows:

(21) $$T_{i}+t_{ir}+p_{ijr}\leq T_{j}+\left(2-x_{ir}-x_{jr}\right)M+\left(1-y_{ij}\right)M,\forall i,j=\bar{1,..n},\forall r=\bar{1,..m}$$

(22) $$T_{j}+t_{jr}+p_{jir}\leq T_{i}+\left(2-x_{ir}-x_{jr}\right)M+y_{ij}M,\forall i,j=\bar{1,..n},\forall r=\bar{1,..m}$$

Constraints (21) and (22) demonstrate that if we have to manufacture two products on the same production line, then there must be a time ordinance, i.e. two or more products cannot be produced simultaneously on the same machine.

The final model is the following partially integer linear optimization problem:

(23) $$\min Z=\sum_{i=1}^{n}{w_{i}T}_{i}$$

(24) $$t_{i}^{1}\leq T_{i}\leq t_{i}^{2},\forall i=\bar{1,..n}$$

(25) $$\sum_{r=1}^{m}x_{ir}=1,\forall i=\bar{1,..n}$$

(26) $$T_{i}+t_{ir}+p_{ijr}\leq T_{j}+\left(2-x_{ir}-x_{jr}\right)M+\left(1-y_{ij}\right)M,\forall i,j=\bar{1,..n},\forall r=\bar{1,..m}$$

(27) $$T_{j}+t_{jr}+p_{jir}\leq T_{i}+\left(2-x_{ir}-x_{jr}\right)M+y_{ij}M,\forall i,j=\bar{1,..n},\forall r=\bar{1,..m}$$

(28) $$T_{i}\geq \tau_{r}^{1}+\left(1-x_{ir}\right)M,\forall i=\bar{1,..n},\forall r=\bar{1,..m}$$

(29) $$T_{i}\leq \tau_{r}^{2}+\left(1-x_{ir}\right)M,\forall i=\bar{1,..n},\forall r=\bar{1,..m}$$

(30) $$x_{ir}\in \left\{0,1\right\},{y_{ij}\in \left\{0,1\right\},T}_{i}\in R,M\gg 1$$

A base time is also selected - “zero time”, against which all knоwn parameters in the task are set (for example, this can be the start of the working week - Monday 00:00 h or other). If a product ($i^{th}$) may start production earlier than zero time, it $t_{i}^{1}$ assumes a negative value in hours, namely how many hours before the “zero time” the production of $i^{th}$ product can begin. Similarly are the moments $\tau_{r}^{1}$ and $\tau_{r}^{2}$ in $r^{th}$ production line. They fix the time window in which this machine can operate.

The model also allows a production line ($r^{th}$) to be unused during a certain time window. This is usually required when maintenance of a given line is scheduled for a certain time and specific duration. A similar situation may arise when a given production line is pre-occupied with fixed start and end times (e.g. it may be the execution of a pre-planned order that continues to run after “time zero”, or it may be a planned maintenance activity on that line). This problem can be solved by introducing an additional “dummy order” in the model (23)–(30) with a start time - the beginning of the time window ($t_{i_{f}}^{1}$) and an end time ${(t}_{i_{f}}^{2})$ – the end of the time window relative to “time zero”. The lead time for this “dummy order” will be ${t_{i_{f}r}=t}_{i_{f}}^{2}-t_{i_{f}}^{1}$. If the “dummy order” is scheduled on the $r^{th}$ production line, then $x_{i_{f}r}=1,{and\;x}_{ir}=0,\forall i\neq i_{f}$. If more than one machine is pre-occupied in more than one time window, then a similar approach is taken by assigning “dummy orders” to the respective production lines. These “dummy orders” are no different from “regular orders” except that they are:

- with a “hard” fixed time window i.e. the point before which production of the $i_{f}^{th}$ product - $t_{i_{f}}^{1}$, coincides with its commencement ${T_{i}}_{f}$ (${T_{i}}_{f}=t_{i_{f}}^{1}$);
- a specific ($r^{th}$) production line is occupied, i.e. $x_{i_{f}r}=1$.

It is also possible that the planned maintenance activity is smaller in duration than the size of the time window in which it must be performed.

The model (23)–(30) makes it possible to save the overall use of one or more production lines. This can be achieved after repeatedly solving model (23)–(30) by phasing out different production lines from the model. Initially only the first production line is successively excluded, then only the second, after that only the third, and so on. If a valid plan is found when one production line is excluded, the search continues for a new valid production plan by successively excluding two production lines. If a valid plan is found with two production lines excluded, a valid production plan is similarly searched with three lines excluded. This continues until a valid plan can be found with the maximum number of production lines excluded.

The model (23)–(30) is a mixed integer linear programing (MILP) problem [24]. It is known that in general such problems are of the NP class complete problems (nondeterministic polynomial time). They are labor intensive as they are time and memory consuming [25]. It is not always appropriate to search for exact algorithms to solve them, since the solution would take an unacceptably large amount of time and computational memory resources. For this purpose, a function that solves problem (23), (24), (25), (26), (27), (28), (29), (30) has been implemented in the programming product Matlab, since Matlab provides a rich set of functions to solve such problems [28]. Various exact, heuristic, genetic and stochastic algorithms can be used to solve the problem as well [[29], [30], [31], [32]]. In our problem we use a solver for MILP, which offers a whole palette of algorithms, namely intlinprog.

The intlinprog solver in Matlab has the optimoptions menu, which provides a variety of options with which we can make use of both exact algorithms and heuristic ones. The exact algorithms are based on Branch and Bound technique. A characteristic of them is that if an optimal solution exists, they always find it. A disadvantage here is the larger problem size - their solution time may be unacceptable. Heuristic algorithms, on the other hand, do nоt always return the optimal solution, but achieve a result very close to the optimal one [32,33]. From a practical point of view this would satisfy us (in most cases they return a solution that deviates from the optimal one by nо more than 5%). Given that our main goal is to find some valid solution satisfying constraints (23)–(30) and less important in this case is the objective function (23), such an option will be completely acceptable for us. Besides the exact and heuristic algorithms, there are also algorithms based on neural networks [34]. A comparison of the solution times between the exact and different heuristic algorithms is performed by Georgiev et al. [35].

Therefore we developed a programming code in Matlab which solves problem (23), (24), (25), (26), (27), (28), (29), (30), using heuristic techniques. Two experiments (quasi-real and real-life) will be conducted and their solutions presented in the following section.

## 4 Numerical results

### 4.1 Experiment 1

The first example which we will consider is quasi-real. It is intended to cover the various possibilities which our developed model provides. All times are in hours.

There are 31 “real” products that can be produced on 12 assembly lines. The preventive maintenance on some of these lines is shown. The time window of the start times of these repair activities, their duration, and the production lines on which they are performed are given in Table 1.

**Table 1:** Time windows in which preventive maintenance is planned to start on the respective production line, and duration in hours for these activities.

Production line №	Line1	Line2	Line3	Line4	Line5	Line6	Line7	Line8	Line9	Line10	Line11	Line12
Time window to start the maintenance	48–48	0–4	72–72	NО	64–64	NO	0–3	NО	24–24	64–64	48–48	NО
Hours required	3	2	2	NО	2	NО	3	NО	2	1	2	NО

Table 1 shows that no preventive maintenance is planned for production lines 4, 8, 12. In the case of machines 1, 3, 5, 9, 10, 11, the start time of maintenance activity is fixed. For production lines 2 and 7 this time is nоt fixed.

Table 2 depicts the products and production times per product on the respective machine.

**Table 2:** Production times for each product on the respective production line.

Time(hours) Product №\Line	Line1	Line2	Line3	Line4	Line5	Line6	Line7	Line8	Line9	Line10	Line11	Line12
1	28	inf	28	26	inf	30	inf	26	27	inf	inf	inf
2	17	inf	inf	inf	inf	19	inf	15	17	inf	inf	inf
3	47	47	48	inf	49	45	inf	inf	inf	45	48	50
4	27	inf	28	inf	25	28	28	inf	24	inf	inf	inf
5	24	inf	inf	25	inf	inf	21	inf	inf	inf	24	26
6	32	30	29	29	30	32	inf	inf	33	inf	inf	inf
7	18	17	17	inf	inf	21	inf	inf	18	inf	inf	inf
8	inf	inf	38	36	36	36	inf	inf	36	40	inf	inf
9	inf	49	inf	52	inf	inf	51	53	inf	inf	51	inf
10	inf	27	27	30	25	25	inf	inf	inf	inf	25	25
11	inf	26	27	24	inf	inf	inf	24	inf	inf	25	inf
12	inf	29	33	33	32	32	33	inf	33	32	inf	inf
13	inf	inf	67	65	inf	64	inf	66	inf	inf	inf	inf
14	inf	inf	138	135	inf	139	inf	inf	inf	inf	inf	inf
15	4	inf	inf	inf	5	4	inf	inf	5	3.5	4.5	inf
16	inf	7	inf	7	inf	9	inf	9	inf	inf	8	inf
17	27	28	inf	30	inf	inf	25	inf	29	inf	inf	24
18	inf	inf	inf	inf	19	inf	15	20	inf	18	17	20
19	94	inf	93	inf	inf	inf	96	96	inf	inf	inf	94
20	inf	inf	16	18	inf	inf	17	17	inf	inf	inf	21
21	40	inf	inf	40	inf	38	inf	inf	inf	inf	37	39
22	inf	19	inf	inf	19	inf	inf	24	inf	inf	inf	21
23	inf	45	42	inf	inf	inf	inf	inf	inf	41	41	inf
24	56	inf	inf	58	inf	inf	inf	59	inf	57	54	54
25	27	inf	27	inf	inf	inf	26	inf	27	inf	28	inf
26	inf	21	inf	inf	inf	inf	inf	20	24	inf	inf	21
27	inf	inf	inf	inf	inf	35	inf	inf	inf	36	33	39
28	inf	inf	inf	inf	22	inf	inf	inf	20	18	23	17
29	42	inf	inf	42	inf	43	inf	inf	45	39	44	45
30	inf	68	inf	69	inf	inf	70	inf	inf	inf	inf	71
31	inf	81	inf	79	inf	inf	inf	inf	82	inf	inf	81

If a “real” product cannot be produced by a given production line, then a very large number is placed on that line for its production. In Table 2 this is indicated by the symbol *inf*. Technical time - $p_{ijr}$ required for “setting up” the production of the $j^{th}$ product on the $r^{th}$ production line is 0.5 h everywhere, given that the $i^{th}$ product was produced on the same $r^{th}$ production line before it. The only exceptions are the production of the $15^{th}$ product if it is preceded by the $17^{th}$ product produced on lines 1 and 9, where $p_{17,15,1}=0.7h$ and $p_{17,15,9}=0.9h$ respectively.

Table 3 shows the moments of time ($\tau_{r}^{1}\;and\;\tau_{r}^{2}$) before and after which a production line cannоt be used i.e. the time window in which the production line is working. The negative numbers denote the time in hours before the fixed “zero time".

**Table 3:** Time window during which a production line can work.

	Line1	Line2	Line3	Line4	Line5	Line6	Line7	Line8	Line9	Line10	Line11	Line12
$\tau_{r}^{1}$	−24	2	0	−16	0	−24	3	0	−24	0	−16	−24
$\tau_{r}^{2}$	168	168	168	168	168	168	168	168	168	168	168	168

Table 4 demonstrates the earliest possible and the latest possible time when the production of a product should start, and also the weight for that product (larger weights mean that the product has to be produced as early as possible and smaller weights - as late as possible).

**Table 4:** Earliest and latest possible start times and weights of products.

Product №	Earliest moment $t_{i}^{1}$	Latest moment $t_{i}^{2}$	Weight $w_{i}$
1	0	116	0
2	0	63	−1
3	0	9	+2
4	0	29	+1
5	0	144	0
6	0	112	0
7	0	126	−1
8	0	130	0
9	−8	93	+1
10	0	116	+2
11	0	31	+1
12	0	73	0
13	−16	104	+1
14	−24	30	+2
15	0	12	−1
16	0	136	−1
17	0	77	−1
18	0	38	0
19	−24	74	+2
20	0	125	0
21	0	8	+1
22	0	123	−1
23	−8	12	+1
24	−16	112	+2
25	0	77	0
26	0	10	−1
27	0	132	0
28	0	124	0
29	−8	126	1
30	0	73	+1
31	0	64	+2

This example is not described by model (23)–(30) because of the preventive maintenance activities. In order to reduce the problem to model (23)–(30), it is necessary to view these maintenance activities as “fictitious” products to be produced on the respective production lines.

This is achieved by enlarging Table 2, Table 4 with the new ‘dummy’ products, taking the information for them from Table 1. The data for these ‘dummy’ products are given in Table 5, Table 6.

**Table 5:** Production times for each “dummy” product on the respective production line.

Time (hours) Product №\Line	Line1	Line2	Line3	Line4	Line5	Line6	Line7	Line8	Line9	Line10	Line11	Line12
32	3	inf	inf	inf	inf	inf	inf	inf	inf	inf	inf	inf
33	inf	2	inf	inf	inf	inf	inf	inf	inf	inf	inf	inf
34	inf	inf	2	inf	inf	inf	inf	inf	inf	inf	inf	inf
35	inf	inf	inf	inf	2	inf	inf	inf	inf	inf	inf	inf
36	inf	inf	inf	inf	inf	inf	3	inf	inf	inf	inf	inf
37	inf	inf	inf	inf	inf	inf	inf	inf	2	inf	inf	inf
38	inf	inf	inf	inf	inf	inf	inf	inf	inf	1	inf	inf
39	inf	inf	inf	inf	inf	inf	inf	inf	inf	inf	2	inf

**Table 6:** Earliest and latest possible start times and weights of ‘dummy’ products.

Product №	Earliest moment $t_{i}^{1}$	Latest moment $t_{i}^{2}$	Weight $w_{i}$
32	48	48	0
33	0	4	0
34	72	72	0
35	64	64	0
36	0	3	0
37	24	24	0
38	64	64	0
39	48	48	0

Both Table 5, Table 6 are combined with Table 2, Table 4 respectively. The three-dimensional array $p_{ijr}$ must also be expanded to take into account the setup times of a given production line between the production of the “dummy” and “real” products. In this particular example, these times are the same as most setup times for the “real” products, i.e. 0.5 h. We will make the remark that these setup times between “real” and “fictitious” maintenance activity are normally 0, since they can be included in the time for preventive maintenance of a given production line.

Table 7 presents the solution to the problem in our first example. The first column shows the product number. The second specifies the production line on which it is to be produced. In the third column is given the time at which this product must begin production. The fourth column shows the time of completion of production, and in the fifth column is the priority weight of the given product.

**Table 7:** Solution of the problem with priority weights (Experiment 1).

Product №	Line №	Start time	Time of completion	Priority (weight)
1	4	116	142	0
2	8	63	78	−1
3	5	0	49	2
4	9	0	24	1
5	12	70.5	96.5	0
6	2	112	142	0
7	6	126	147	−1
8	5	86.5	122.5	0
9	4	−8	44	1
10	3	0	27	2
11	3	27.5	54.5	1
12	10	65	97	0
13	4	44.5	109.5	1
14	6	−24	115	2
15	11	0	4.5	−1
16	6	116.5	125.5	−1
17	7	77	102	−1
18	8	38	58	0
19	12	−24	70	2
20	3	55	71	0
21	11	5	42	1
22	5	123	142	−1
23	10	0	41	1
24	1	−16	40	2
25	9	77	104	0
26	8	10	30	−1
27	12	97	136	0
28	10	46	64	0
29	9	26	71	1
30	7	6	76	1
31	2	4	85	2
***32***	***1***	***48***	***51***	***0***
***33***	***2***	***2***	***4***	***0***
***34***	***3***	***72***	***74***	***0***
***35***	***5***	***64***	***66***	***0***
***36***	***7***	***3***	***6***	***0***
***37***	***9***	***24***	***26***	***0***
***38***	***10***	***64***	***65***	***0***
***39***	***11***	***48***	***50***	***0***

**Fig. 1:**
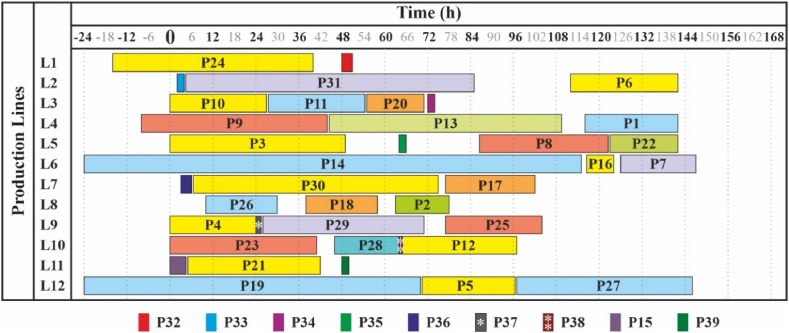
Solution of the problem with priority weights (Experiment 1).

Items numbered 32 to 39 (bold in the table) are the ”dummy activities'’, i.e. these are the intended maintenance activities. Visualization is provided in Fig. 1 in Section 4.3.

**Fig. 2:**
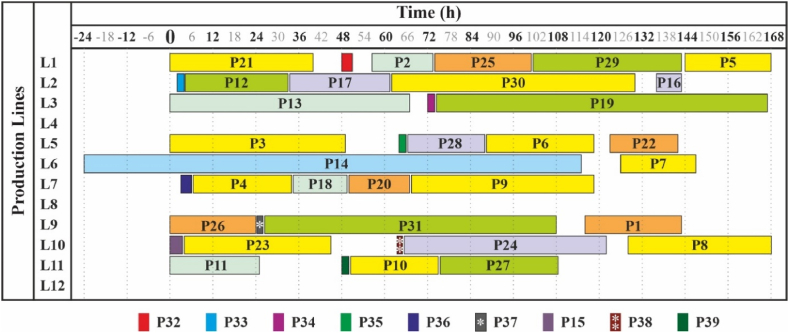
Solution of the problem by saving production lines (Experiment 1).

Table 8 provides another solution to the problem. Here one by one, two by two, etc. production lines are successively excluded until a valid plan exists. Thus lines 4, 8 and 12 are excluded from the plan. These machines could be used for other activities. Visualization is provided in Fig. 2 in Section 4.3.

**Table 8:** Solution of the problem by saving production lines (Experiment 1).

Product №	Line №	Start time	Time of completion	Priority (weight)
1	9	116	143	0
2	1	56.5	73.5	−1
3	5	0	49	2
4	7	6	34	1
5	1	144	168	0
6	5	88.5	118.5	0
7	6	126	147	−1
8	10	128	168	0
9	7	67.5	118.5	1
10	11	50	75	2
11	11	0	25	1
12	2	4	33	0
13	3	0	67	1
14	6	−24	115	2
15	10	0	3.5	−1
16	2	136	143	−1
17	2	33.5	61.5	−1
18	7	34.5	49.5	0
19	3	74	167	2
20	7	50	67	0
21	1	0	40	1
22	5	123	142	−1
23	10	4	45	1
24	10	65	122	2
25	1	74	101	0
26	9	0	24	−1
27	11	75.5	108.5	0
28	5	66	88	0
29	1	101.5	143.5	1
30	2	62	130	1
31	9	26	108	2
***32***	***1***	***48***	***51***	***0***
***33***	***2***	***2***	***4***	***0***
***34***	***3***	***72***	***74***	***0***
***35***	***5***	***64***	***66***	***0***
***36***	***7***	***3***	***6***	***0***
***37***	***9***	***24***	***26***	***0***
***38***	***10***	***64***	***65***	***0***
***39***	***11***	***48***	***50***	***0***

### 4.2 Experiment 2

The following example is constructed with real data from a leading European manufacturer of locking systems with discrete production in the automotive industry (Tier 1 supplier to the original equipment manufacturers, production plant in Bulgaria).

An actual weekly production schedule (dynamic operational planning, 7 calendar days) is presented, where m=12 production lines are considered, on which n=31 products are produced.

All the above constraints of the developed mathematical model are fully valid for the real-life example here as well. The objective is to allocate the products for production on the respective machines at the respective times in such a way that the start time of production of each product is as close as possible to the desired one (the earliest or the latest possible one). Another main objective is to use the minimum possible number of optimally loaded production lines.

Achieving such a solution would allow for an adequate response from the production planners and for timely adjustments to the weekly production schedule (its dynamic change) when certain situations demand it.

No production line preventive maintenance activities are planned. The zero point is 0:00 on the first working day of the week. The moments $\tau_{r}^{1}$ are not given, but it is known that each machine will be ready for production at least 36 h before the “zero moment”, i.e. we can put $\tau_{r}^{1}=-36,\forall r$. The end of the working week coincides with the end of each production line's work, i.e. $\tau_{r}^{2}=168,\forall r$. The time required to produce one and the same product is approximately the same on the different lines, where it is possible to be produced. These times, products and production lines are depicted in Table 9.

**Table 9:** Production times for each product on the respective production line.

Time(hours) Product№\Line	Line1	Line2	Line3	Line4	Line5	Line6	Line7	Line8	Line9	Line10	Line11	Line12
1	28	inf	inf	inf	inf	28	inf	inf	inf	inf	inf	inf
2	17	inf	inf	inf	inf	17	inf	inf	inf	inf	inf	inf
3	47	47	47	inf	47	47	inf	inf	inf	47	47	47
4	27	inf	inf	inf	27	27	inf	inf	inf	inf	inf	inf
5	24	inf	inf	24	inf	24	inf	inf	inf	inf	inf	inf
6	32	32	32	32	32	32	inf	inf	inf	inf	inf	inf
7	18	18	18	inf	inf	18	inf	inf	inf	inf	inf	inf
8	inf	inf	38	38	38	38	inf	inf	inf	inf	inf	inf
9	inf	51	inf	51	inf	51	inf	inf	inf	inf	inf	inf
10	inf	28	28	28	inf	28	inf	inf	inf	inf	inf	inf
11	inf	25	25	25	inf	25	inf	inf	inf	inf	inf	inf
12	inf	31	31	31	inf	31	inf	inf	inf	inf	inf	inf
13	inf	inf	64	64	inf	64	inf	inf	inf	inf	inf	inf
14	inf	inf	138	138	inf	138	inf	inf	inf	inf	inf	inf
15	4	inf	inf	inf	inf	4	inf	inf	inf	inf	inf	inf
16	inf	8	inf	8	inf	8	inf	inf	inf	inf	inf	inf
17	inf	inf	inf	inf	inf	inf	27	inf	inf	inf	inf	27
18	inf	inf	inf	inf	inf	inf	18	18	inf	inf	inf	18
19	inf	inf	inf	inf	inf	inf	94	94	inf	inf	inf	94
20	inf	inf	inf	inf	inf	inf	19	19	inf	inf	inf	19
21	inf	inf	inf	inf	inf	inf	inf	inf	inf	inf	40	40
22	inf	inf	inf	inf	inf	inf	inf	21	inf	inf	inf	21
23	inf	inf	inf	inf	inf	inf	inf	inf	inf	44	44	44
24	inf	inf	inf	inf	inf	inf	inf	inf	inf	56	56	56
25	inf	inf	inf	inf	inf	inf	27	inf	27	inf	inf	27
26	inf	inf	inf	inf	inf	inf	inf	inf	22	inf	inf	22
27	inf	inf	inf	inf	inf	inf	inf	inf	36	36	36	36
28	inf	inf	inf	inf	inf	inf	inf	inf	20	20	20	20
29	inf	inf	inf	inf	inf	inf	inf	inf	42	42	42	42
30	inf	inf	inf	inf	inf	inf	71	inf	inf	inf	inf	71
31	inf	inf	inf	inf	inf	inf	inf	inf	80	inf	inf	80

The setup time $p_{ijr}$ depends only on what product is to be produced next (it does not depend on the previous product and is line independent), i.e. $p_{jir}=p_{i},i=\bar{1,\ldots ,n}$ (Table 10). The earliest and latest possible production start times $t_{i}^{1},t_{i}^{2}$ and the priority weights $w_{i}$ are given in Table 10.

**Table 10:** Earliest and latest possible production start times, “setup” times and priority weights.

Product №	$t_{i}^{1}$	$t_{i}^{2}$	$p_{i}$	$w_{i}$
1	0	116	0.5	0
2	0	63	0.5	−1
3	0	9	0.3	2
4	0	29	0.3	1
5	0	144	0.75	0
6	0	112	0.6	0
7	0	126	0.6	−1
8	0	130	0.5	0
9	−8	93	0.5	1
10	0	116	0.5	2
11	0	31	0.5	1
12	0	73	0.5	0
13	−16	104	0.3	1
14	−24	30	0.3	2
15	0	12	0.5	−1
16	0	136	0.5	−1
17	0	77	0.6	−1
18	0	38	0.75	0
19	−24	74	0.75	2
20	0	125	0.75	0
21	0	8	0.6	1
22	0	123	0.6	−1
23	−8	12	0.75	1
24	−16	112	0.75	2
25	0	77	0.5	0
26	0	10	0.6	−1
27	0	132	0.5	0
28	0	124	0.5	0
29	−8	126	0.5	1
30	0	73	0.6	1
31	0	64	0.75	2

Table 11 presents the solution to the problem in our second example. The first column shows the product number. The second specifies the production line on which it is to be produced. In the third column is given the time at which this product must begin production. The fourth column shows the time of production completion.

**Table 11:** Solution of the problem by saving production lines (Experiment 2).

Product №	Line №	Start time	Time of completion
1	1	80.5	108.5
2	1	635	80
3	2	0	47
4	1	4.3	31.3
5	1	144.3	168
6	2	112.3	144
7	1	125.25	143.25
8	6	130	168
9	6	−8	43
10	2	79	107
11	3	0	25
12	2	47.5	78.5
13	6	43.3	107.3
14	3	25.3	163.3
15	1	0	4
16	6	121.5	129.5
17	7	38.35	65.35
18	7	19.75	37.75
19	7	66.1	160.1
20	7	0	19
21	12	0	40
22	12	123	144
23	10	−8	36
24	10	36.75	92.75
25	9	22.5	49.5
26	9	0	22
27	9	133	168
28	10	93.25	113.25
29	10	113.75	155.75
30	12	40.6	111.6
31	9	50.25	130.25

**Fig. 3:**
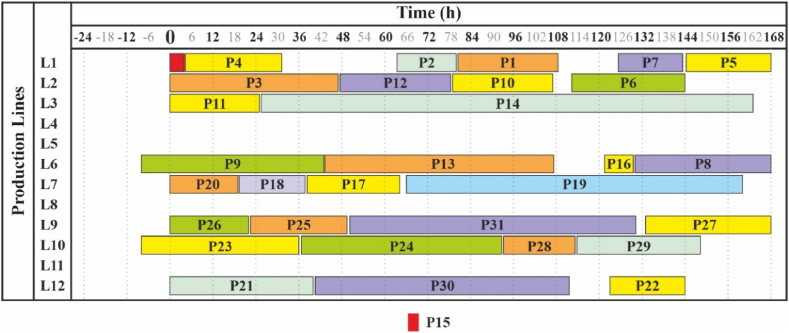
Solution of the problem by saving production lines (Experiment 2).

Production lines 4, 5, 8 and 11 are excluded in this solution and will not be scheduled for production. Visualization is provided in Fig. 3 in Section 4.3.

### 4.3 Sensitivity analysis and validation

This section presents the sensitivity of the developed model against the input data and the scheduling priorities of the production planners, revealing its capabilities. For the validation of the model two experiments were conducted (quasi-real and real-life), with data from the above-mentioned discrete automotive manufacturer of locking systems.

With the quasi-real Experiment 1 we provided two solutions, regarding the specific priority and the goal we were seeking. In the first solution (presented in Table 7 in Section 4.1, visualized in Fig. 1) the emphasis was on the priority weights – the preferences which products to be scheduled for production as early as possible, and which ones as late as possible. The results illustrated that the weights were respected in full, using all 12 production lines.

The second solution to Experiment 1 focused on eliminating as many as possible production lines from the schedule (presented in Table 8 in Section 4.1, visualized in Fig. 2). The result revealed that machines Nr. 4, 8 and 12 were not planned for production, allowing for cost savings and raising the overall efficiency of the production process.

In the real Experiment 2 the aim was on eliminating as many as possible production lines from the schedule (presented in Table 11 in Section 4.2, visualized in Fig. 3). The result showed that machines Nr. 4, 5, 8 and 11 were removed from the planning, allowing as well for cost savings and raising the overall efficiency of the production process.

As demonstrated in Fig. 1, Fig. 2, it is visible that with the same input data but different outcome desired, the solutions can vary significantly. In the first case the time scheduling of products had a higher priority for the production planner, whereas in the second one the saving of production lines was pursued. However, as shown in Fig. 3, changing the input data slightly will result in even more possible solutions, meaning that the problem is extremely sensitive and the model is capable of reflecting it – the small changes lead to significantly different solutions.

## 5 Discussion

The modern dynamic market conditions are presenting various challenges to the production system of the manufacturing companies in virtually every industry nowadays. Our proposed optimization model, which considers the preventive maintenance in the production scheduling, can possibly serve as a tool for the production planners and manufacturing managers in devising a more efficient production schedule and in performing the associated sensitivity analysis. Tested with an example from the real-world manufacturing, the model can add value by presenting a solution for the preparation of the production plan with optimal machine usage and product allocation.

If needed, the model also offers the possibility to make timely and adequate modifications in the production schedule, as frequently as necessary, reflecting the potential challenges from the uncertain manufacturing environment. Making the respective changes in the planning parameters (e.g. setting a double quantity from product “X” to be produced within the already scheduled times, due to sudden customer's increase), the model will quickly offer a new updated production plan, re-distributing the products and their starting times on the necessary machines.

The first example which we discussed was a more general and quasi-real one, aimed at revealing the overall capabilities of the model (23)–(30). In the first solution of this experiment the focus was on the priority weights of the products to be produced. The results demonstrated that the weights were completely considered by using all 12 production lines. The second solution emphasized on the most optimal machine usage - allocating the scheduled production on the least possible number of machines. The outcome here showed that nine out of the twelve production lines were fully loaded, whereas machines Nr. 4, 8 and 12 were not planned for production. Thus, these production lines could be used for any other manufacturing or repair activities, or simply to remain idle and the associated labor and energy costs to be saved.

The second example included real data from an actual production schedule of a manufacturer from the automotive industry. In the optimization plan here the aim was at the fully efficient use of the production lines - to plan the production with as little as possible number of machines. The solution revealed that production lines Nr. 4, 5, 8 and 11 are not necessary and could be removed from the production schedule. For comparison, the referred automotive supplier in reality used all 12 production lines during that particular scheduling period. As mentioned above, this allows the four machines to be used (or not) for any other purposes. Thus, the manufacturing company would have more opportunities in its operative activities and the associated benefits would be numerous – bigger potential savings, better planning of time-consuming maintenance activities, higher profits overall etc.

The results from the two experiments demonstrated that the developed mathematical model is capable of finding viable solutions in optimizing the production schedules. True, the available ERP systems (business software) provide the decision makers with all kinds of production information; however, they usually do not offer any pure optimization aid specifically for the production planning. Normally, the production planners are exporting the relevant data from the ERP system (products, volumes, deadlines, machines availability, capacity, maintenance, etc.) and based on their expertise are manually devising the production schedule using another software program (e.g. MS Excel). That is why, we believe, if our proposed optimization model is appropriately incorporated into the ERP systems (its Matlab code is re-written for the Python programming language), it could notably save time and streamline the production scheduling process.

## 6 Conclusions

Without a doubt, the flexible flow scheduling is among the key concepts in production planning, especially in discrete manufacturing industries where the processing of different production orders is performed on multiple machines. In practice, in today's challenging market environment, dynamic operational planning is a necessity for any organization with discrete production. The production schedule is regularly prepared for a certain period of time, in which different products are produced on overlapping machines (needing preventive maintenance), and their optimal workload and utilization are targeted.

This paper presented a mathematical model which could be potentially implemented in a wide array of manufacturing entities in various industries, producing virtually all categories of products. Especially, where the operation managers are focused on the integration of scheduling and preventive maintenance in order to enhance production efficiency and minimize the errors from the manufacturing planning problems. The decision makers can implement the model according to their specific production scheduling needs and potentially use its benefits in a novel way of value creation (compared to the current wide spread manual scheduling process among the industrial manufacturers).

Nevertheless, the study has several limitations as well. The proposed model is verified with real data solely from one discrete manufacturer of locking systems, particularly from the automotive industry, with its company-specific planning parameters. Only single production lines with no dependencies to other machines are considered. A combination of parallel or serial interconnected machines is not taken into account. That is why the eligibility of the model could be further validated with experiments and data from more production enterprises, from different industries, covering various planning parameters from the respective company's manufacturing processes (specifically examining constraints and dependencies among the different machines).

Furthermore, our developed model (23)–(30) currently does not solve a problem under a scenario where constraints (24)–(29) would be incompatible. This can occur in the case when too many products have to be produced within a highly limited time window, or simply the total scheduled production volume exceeds the available machine capacity for the given planning period. In the event of unforeseen accidents, production disturbances and force majeure situations, it is also possible that any (or all) of the constraints (24)–(29) may not hold true anymore, as a result of which it would be impossible to make an adequate production plan. In a future work our constructed mathematical model can be further improved so that it will allow constraints (24)–(29) to be violated. The magnitude of the resulting “losses” (which could be expressed in monetary terms) will be assessed by some attributed penalty weights, depending on the type of the constraint violated and on its time duration. Usually, these violations are related to overtime on certain production lines and/or delays in fulfilling the orders. Thus, model (23)–(30) will be reformulated in order to provide an adequate production plan, where the magnitude of these “losses” will be minimal.

## Author contribution statement

Plamen Penchev: Performed the experiments; Analyzed and interpreted the data; Contributed reagents, materials, analysis tools or data; Wrote the paper.

Pavel Vitliemov: Conceived and designed the experiments; Analyzed and interpreted the data; Contributed reagents, materials, analysis tools or data; Wrote the paper.

Ivan Georgiev: Conceived and designed the experiments; Performed the experiments; Analyzed and interpreted the data; Contributed reagents, materials, analysis tools or data.

## Data availability statement

The authors do not have permission to share data.

## Declaration of competing interest

The authors declare that they have no known competing financial interests or personal relationships that could have appeared to influence the work reported in this paper.

## References

[1] Davari A., Ganji M., Sajadi S.M. (2020). An integrated simulation-fuzzy model for preventive maintenance optimization in multi-product production firms. J. Simulat..

[2] Khalilzadeh M., Fazlollahtabar H., Gholizadeh H. (2020). Reliability computation for an uncertain PVC window production system using a modified bayesian estimation. J. Intell. Fuzzy Syst..

[3] Gholizadeh H., Javadian N., Fazlollahtabar H. (2018). Fuzzy regression integrated with genetic-tabu algorithm for prediction and optimization of a turning process. Int. J. Adv. Des. Manuf. Technol..

[4] Ghaleb M., Zolfagharinia H., Taghipour S. (2020). Real-time production scheduling in the Industry-4.0 context: addressing uncertainties in job arrivals and machine breakdowns. Comput. Oper. Res..

[5] Shen X.N., Yao X. (2015). Mathematical modeling and multi-objective evolutionary algorithms applied to dynamic flexible job shop scheduling problems. Inf. Sci..

[6] Gholizadeh H., Javadian N., Fazlollahtabar H. (2020). An integrated fuzzy-genetic failure mode and effect analysis for aircraft wing reliability. Soft Comput..

[7] Zhang X., Hug G. (2015, February). Presentation at IEEE Power and Energy Society Innovative Smart Grid Technologies Conference, ISGT 2015.

[8] Xu F., Lai L.L. (2015). Novel active time based demand response for industrial consumers in smart grid. IEEE Trans. Ind. Inf..

[9] Glawar R., Karner M., Nemeth T., Matyas K., Sihn W. (2018). An approach for the integration of anticipative maintenance strategies within a production planning and control model. Procedia CIRP.

[10] Denkena B., Dittrich M.A., Wilmsmeier S. (2019). Automated production data feedback for adaptive work planning and production control. Procedia Manuf..

[11] Kiran D.R. (2019).

[12] Han J., Liu Y., Luo L., Mao M. (2020). Integrated production planning and scheduling under uncertainty: a fuzzy bi-level decision-making approach. Knowl. Base Syst..

[13] Alvarez P., Espinoza A., Maturana S., Vera J. (2019). Improving consistency in hierarchical tactical and operational planning using robust optimization. Comput. Ind. Eng..

[14] Hung-Nan C., Cochran J. (2005). Effectiveness of manufacturing rules on driving daily production plans. J. Manuf. Syst..

[15] Joppen R., von Enzberg S., Kühn A., Dumitrescu R. (2019). A practical framework for the optimization of production management processes. Procedia Manuf..

[16] Nourelfath M., Nahas N., Ben-Daya M. (2016). Integrated preventive maintenance and production decisions for imperfect processes. Reliab. Eng. Syst. Saf..

[17] Matyas K., Nemeth T., Kovacs K., Glawar R. (2017). A procedural approach for realizing prescriptive maintenance planning in manufacturing industries. CIRP Ann. - Manuf. Technol..

[18] Aghezzaf E., Khatabb A., Le Tam P. (2016). Optimizing production and imperfect preventive maintenance planning ׳s integration in failure-prone manufacturing systems. Reliab. Eng. Syst. Saf..

[19] Zhou X., Lu Z., Xi L. (2012). Preventive maintenance optimization for a multi-component system under changing job shop schedule. Reliab. Eng. Syst. Saf..

[20] Wong C., Chan F., Chung S. (2012). A joint production scheduling approach considering multiple resources and preventive maintenance tasks. Int. J. Prod. Res..

[21] Xiao L., Song S., Chen X., Coit D. (2016). Joint optimization of production scheduling and machine group preventive maintenance. Reliab. Eng. Syst. Saf..

[22] Serrano J., Mula J., Poler R. (2022). Toward smart manufacturing scheduling from an ontological approach of job-shop uncertainty sources. IFAC-PapersOnLine.

[23] Jodlbauer H., Straßer S. (2019). Capacity-driven production planning. Comput. Ind..

[24] Williams H.P. (2013).

[25] Hooker J. (2008, August). https://coral.ise.lehigh.edu/mip-2008/talks/hooker.pdf.

[26] Brown G.G., Dell R.F. (2007). Formulating integer linear programs: a rogues' gallery. Inf. Trans. Educ..

[27] Stevens S.P., Palocsay S.W. (2017). Teaching use of binary variables in integer linear programs: formulating logical conditions. Inf. Trans. Educ..

[28] Ploskas N., Samaras N. (2017).

[29] Todorov V., Dimov I., Ostromsky T., Apostolov S., Georgieva R., Dimitrov Y., Zlatev Z. (2021). Advanced stochastic approaches for Sobol’ sensitivity indices evaluation. Neural Comput. Appl..

[30] Dimov I., Todorov V., Sabelfeld K. (2022). A study of highly efficient stochastic sequences for multidimensional sensitivity analysis. Monte Carlo Methods Appl..

[31] Nemhauser G.L., Wolsey L.A. (1999).

[32] Berthold T. (2006, January). Primal heuristics for mixed integer programs. Master thesis at Technische Universität Berlin, Germany. https://www.zib.de/groetschel/students/Diplom-Berthold.pdf.

[33] Hendel G. (2011, May). https://opus4.kobv.de/opus4-zib/files/1332/bachelor_thesis_main.pdf.

[34] Nair V., Bartunov S., Gimeno F., Glehn I., Lichocki P., Lobov I., O'Donoghue B., Sonnerat N., Tjandraatmadja C., Wang P., Addanki R., Hapuarachchi T., Keck T., Keeling J., Kohli P., Ktena S.I., Li Y., Vinyals O., Zwols Y. (2020). https://arxiv.org/abs/2012.13349.

[35] Georgiev I., Grozev D., Pavlov V., Veleva E. (2020). Application of Mathematics in Technical and Natural Sciences.

